# Experimental Study of Hydrogasification of Lignite and Subbituminous Coal Chars

**DOI:** 10.1155/2015/867030

**Published:** 2015-05-06

**Authors:** Stanisław Gil, Adam Smoliński

**Affiliations:** ^1^Institute of Metals Technology, Silesian University of Technology, Krasińskiego 8, 40-019 Katowice, Poland; ^2^Department of Energy Saving and Air Protection, Central Mining Institute, Plac Gwarków 1, 40-166 Katowice, Poland

## Abstract

The experimental facility for pressure hydrogasification research was adapted to the pressure of 10 MPa and temperature of 1300 K, which ensured repeatability of results and hydrogen heating to the process temperature. A hydrogasification reaction of chars produced from two rank coals was investigated at temperatures up to 1173 K, pressures up to 8 MPa, and the gas flow rates of 0.5–5 dm_n_^3^/min. Reactivity of the “Szczerców” lignite char was found to be slightly higher than that of the subbituminous “Janina” coal char produced under the same conditions. A high value of the char reactivity was observed to a certain carbon conversion degree, above which a sharp drop took place. It was shown that, to achieve proper carbon conversion, the hydrogasification reaction must proceed at a temperature above 1200 K.

## 1. Introduction

Coal gasification involves carbon conversion in the presence of gasifying medium (oxygen, hydrogen, water steam, carbon dioxide, or others) into gaseous products which, following suitable processing, are used for chemical and/or energy industry purposes as a synthesis gas.

Placing coal in the hydrogen atmosphere at increased temperature and pressure results in a two-stage process: (a) hydropyrolysis where volatile matter released from coal reacts with hydrogen to produce primary, methane-rich gas as well as liquid products and solid char, which is highly reactive during the initial stage, (b) hydrogasification of the char of a high initial reactivity which decreases with the gasification degree. For the char, the hydrogasification rate is the highest immediately after coal devolatilisation and it decreases with the gasification degree accordingly to a proposed by Johnson [1] exponential function, which was expanded by Mühlen [2] to a char gasification degree- and process temperature-dependent relationship.

Hydrogasification is an exothermal reaction between hydrogen and carbon

(1) $$\begin{array}{c} \text{C}+2{\text{H}}_{2}⟶\text{C}{\text{H}}_{4}+74.9\;\text{kJ}/\text{kmol} \end{array}$$

where the reaction product is methane. The principles of hydrodynamic equilibrium in the H_2_-CH_4_-C system suggest that, to obtain a high molar fraction of methane in the resulting H_2_-CH_4_ mixture, the hydrogasification process must be performed at high pressure. A kinetic mechanism of hydrogasification, where a term of free active centres is applied, was proposed by Blackwood [3, 4].

Mühlen [2] studied the effects of pressure on the process rate during coal char gasification in the H_2_, H_2_O, and CO_2_ atmospheres. He presented a comparison of the process rates for the three types of char gasification under *T* = 1173 K isothermal conditions as a pressure function and the temperature function at *p* = 2 MPa. Mühlen demonstrated that the effect of pressure on the hydrogasification rate was different from that observed during gasification in H_2_O and CO_2_. A characteristic maximum rate of char gasification in H_2_O and CO_2_ at as low pressures as approximately 2 MPa was observed. For the whole pressure range, the gasification rate in H_2_O was more than threefold higher than the gasification rate in CO_2_. For low pressures, hydrogasification was the slowest and it did not reach the gasification rate in CO_2_ until the pressure of approximately 4 MPa was attained. For the gasification rate in H_2_O, a pressure of approximately 10 MPa was necessary. Moreover, hydrogasification rate reduction was observed for higher char conversion rates.

In the initial stage of coal gasification, called hydropyrolysis, hydrogen is essential due to its effect on primary tar and the solid carbon skeleton. Coal pyrolysis in the presence of hydrogen markedly increases carbon conversion to gaseous C_1_–C_3_ hydrocarbons, even at relatively low pressures [5, 6]. Under proper conditions and in the presence of hydrogen, coal pyrolysis products of high molecular mass are mainly conversed to methane. Thus, hydropyrolysis may be considered as a hydrogasification part with very short residence times.

Moseley and Paterson [7] made an attempt to describe coal hydrogasification as simultaneous stages of coal hydropyrolysis and char hydrogasification. Their model was further expanded by Zahradnik and Glenn [8] by applying the concept of active centres, introduced by Blackwood [3] into the mechanism of char hydrogasification.

Anthony et al. [9] studied loss of a coal sample mass during its rapid heating to the final temperature and holding it for 5–20 s under the inert and hydrogen atmospheres. At temperatures above 873 K, hydrogen at 6.9 MPa increased the overall mass loss. For methane, an increased yield was also observed in the hydrogen atmosphere at the temperature above 773 K and the pressure of 6.9 MPa. Suuberg et al. [10] performed similar, and the most frequently cited in the scientific literature, studies on hydropyrolysis.

On the other hand, effects of the atmosphere on amounts of liquid products released at higher pressures are ambiguous. Any comparisons between results obtained by various authors are difficult to perform due to different types of coal used in investigations, differences between experimental devices, and diverse understanding of the term “liquid products” or “tar.” In the literature, descriptions of studies conducted by four independent authors for the same Westerholt coal under the inert and hydrogen atmospheres can be found. In their investigations, Bunthoff [11] and Krabiell [12] used a thermobalance, Kaiser [13] applied the Curie point method, and Treuling [14] devolatilised a relatively large 500 g coal sample in a fixed bed. It should be noted that, in the inert atmosphere, comparable amounts of liquid products per coal mass unit were obtained despite definitely different heating rates varying between 0.05 K/s and 9000 K/s. In the hydrogen atmosphere, on the other hand, differences were significant but there were evidently comparable amounts of liquid products reported in the studies conducted by Kaiser [13] and Treuling [14]. An analysis of qualitatively different curve courses according to Krabiell [12] and Bunthoff [11] requires an in-depth understanding of the definition of the term “liquid products” used by individual researchers.

A major disadvantage of hydrogasification process is the requirement for high temperatures and pressures, which affects the economy of industrial facilities. An application of catalysts is a solution to some problems that occur during hydrogasification as there is a potential for conducting the process at a lower temperature. Certain minerals and metals that show a catalytic effect on hydrogasification reactions are particularly attractive. Alkali metals, due to their high activity and availability, have become a subject of great interest for researchers. The aims of catalyst use are as follows: to increase a carbon conversion rate in the gasification reactor, to reduce a gasification temperature, and to affect selectively a composition of produced gas. The catalytic effect can be observed both in a heterogeneous reaction on the char surface and in a homogeneous reaction in the gaseous phase. The gaseous phase reactions are also affected by fly ash while the char surface reactions are influenced by minerals dispersed in its particles.

Experiments have revealed that catalyst-related advantages are particularly important for these gasification methods where the reaction intensity cannot be enhanced in a conventional way by increasing temperature, for example, direct coal gasification into methane or allothermal gasification using an external, diaphragm heat supply.

Further temperature reduction can be achieved when eutectic salts and their mixtures are applied as catalysts. Because improved catalyst contact with coal results in increased gasification rate, a concept of liquid catalytic salts with the ability of better penetrating macropores in the coal (char) structure that leads to increased carbon conversion is adopted. Thus, eutectic salts with markedly lower melting points than those of pure salts should enhance catalytic gasification at low temperatures.

Sheth et al. [15] presented experiments regarding effects of the H_2_/H_2_O ratio on char conversion time during gasification at 1005 K and 2.64 MPa with a catalyst (43.5% Li_2_CO_3_, 31.5% Na_2_CO_3_, and 25% K_2_CO_3_) mixed in a 10% mass proportion with the Illinois number 6 coal before devolatilisation at 1023 K. The investigations showed an inhibiting effect of hydrogen on the gasification rate. The hydrogasification (H_2_/H_2_O = ∞) was far slower than gasification in the presence of water steam (H_2_/H_2_O = 0). The rate of hydrogasification with this catalyst was 0.0019 per minute and approximately 4-fold higher than the rate of hydrogasification without a catalyst. It should be noted here that the results are comparable to calculations performed by Mühlen [2] for the Fűrst Leopold coal.

The aim of the paper is to present the experimental results of the “Janina” and “Szczerców” coal char hydrogasification and to determine the effects of process parameters, such as pressure, temperature, and reactivity, on coal hydrogasification. In particular, the investigations were focused on a relationship between reactivities of chars and conversion rates, which is technologically important. Moreover, comparative investigations of coal hydrogasification and char gasification in the H_2_ + CO_2_ atmosphere were performed.

Due to difficulties with respect to a coal hydrogasification description, resulting mainly from simultaneous coal hydropyrolysis and hydrogasification of the remaining char, a mathematical model of char hydrogasification (described in [16]) was only developed.

## 2. Materials and Methods

**Figure 1:**
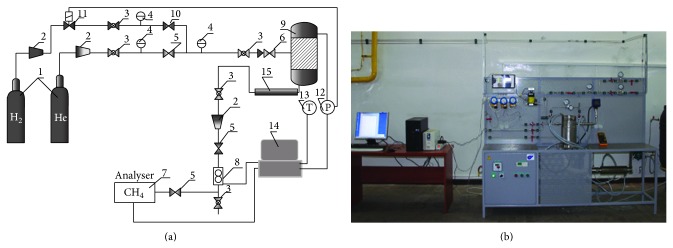
(a) Scheme: 1: gas cylinder, 2: pressure regulator, 3: cut-off value, 4: manometer, 5: control value, 6: return valve, 7: methane analyzer, 8: flow meter, 9: reactor, 10: single stage pressure regulator, 11: electromagnetic valve, 12: pressure converter, 13: thermocouple, 14: computer, 15: cooler and (b) view of the experimental facility.

A schematic diagram of the experimental facility is presented in Figure 1(a). A pressure reactor allows for hydrogasification of up to 30 g samples with the following parameters: a hydrogen flow rate of 0.1–10 dm_n_^3^/min, pressure of up to 10 MPa, and temperature of up to 1273 K. The reactor was equipped with electrical sample heating system (1 K/s) ensuring simultaneous hydrogen heating to the process temperature. When the samples were placed inside the reactor, it was flushed with helium and then filled with hydrogen. The hydrogen flow rate was stabilised (±1.5% deviation) in a mass flow controller.

Measurements of CH_4_ concentration in the process gas were performed using a microprocessor analyser with infrared absorption detectors. An analyser with two MG-73/CH_4_ measuring heads (0–5% and 0–100%) as well as an MSMR-4 microprocessor recording system was used.

The process parameters were recorded in accordance with a set mode of 5 s intervals. A view of the experimental facility is presented in Figure 1(b). A module design of the facility allowed for further expanding the measurement and flow channels of the process gases. It allowed, in particular, for delivering mixtures of hydrogen and an additional reagent to the process.

Calculations for two extreme cases, that is, 973 K and 6 MPa as well as 1273 K and 10 MPa, were performed. For each case, distribution of thermal fields and flows in an empty reactor and in a reactor containing 10 g of coal was considered. While modelling the char-filled reactor, calculations for multiphase flows were necessary and the stationary bed with a solid phase containing mean 0.8 mm particles was approximated. The calculations resulted in flow parameter fields typical of reactors that operate under two extreme experimental conditions, which was ensured by the gas swirler design.

The char was produced during 30 min pyrolysis (heating rate of 100 K/s) in helium at 2 MPa and 1373 K. The char samples were sieved to the group of 0.6–1.0 mm particle size. The “Janina” subbituminous coal and the “Szczerców” lignite were used during the experiments, of the characteristics described in Table 1.

The measured molar methane content was used to calculate the rate of hydrogasification reactions. Because hydrogen was supplied to the reactor, while the measured molar gas stream ${\overset{{}^{\circ}}{n}}_{g}$ leaving the reactor contained both hydrogen and methane, the rate of hydrogasification, $\overset{{}^{\circ}}{R}$, equivalent to the rate of methane produced, was calculated using the equation:

(2) $$\begin{array}{c} \overset{{}^{\circ}}{R}={\overset{{}^{\circ}}{n}}_{g}\text{C}{\text{H}}_{4}. \end{array}$$

The number of carbon moles remaining in the reactor in the solid char at time *t* is as follows:

(3) $$\begin{array}{c} n_{C}\left(t\right)=n_{C}\left(0\right)-\overset{t}{\underset{0}{\int }}{\overset{{}^{\circ}}{n}}_{g}\text{C}{\text{H}}_{4}dt, \end{array}$$

where *n*_*C*_(0) is the initial number of carbon moles in the char sample.

The char hydrogasification experiments were performed on 10 g samples of the “Janina” and “Szczerców” coals. The hydrogasification schedule involved experiments at 973, 1073, and 1173 K; 6, 7, and 8 MPa; and hydrogen flow rate of 0.5, 2, and 5 dm_n_^3^/min in the following series: 5 dm_n_^3^/min; 973–1173 K; 6–8 MPa; 2 dm_n_^3^/min; 973–1173 K; 6–8 MPa; 0.5 dm_n_^3^/min; 973–1173 K; 6–8 MPa. Duration of each experiment was different and determined at the reaction completion.

The “Janina” and “Szczerców” coal hydrogasification schedule involved experiments with 10 g samples at 973 and 1173 K, 8 MPa and hydrogen flow rate of 0.5 dm^3^/min.

The schedule of hydrogasification in the CO_2_-diluted hydrogen for the “Janina” char was followed for a mixture of H_2_+ 10% CO_2_ and involved experiments at 973, 1073, and 1173 K; 8 MPa; hydrogen flow rate of 0.5 dm_n_^3^/min, with 10 g samples. Duration of each experiment was different and determined at the reaction completion. The investigations were performed for the maximum pressure value of 8 MPa and the lowest gas flow rate of 0.5 dm_n_^3^/min as under these conditions, the maximum methane concentration was obtained in the hydrogasification experiments conducted in pure hydrogen.

## 3. Results and Discussion

### 3.1. Carbon Conversion and Methane Content

**Figure 2:**
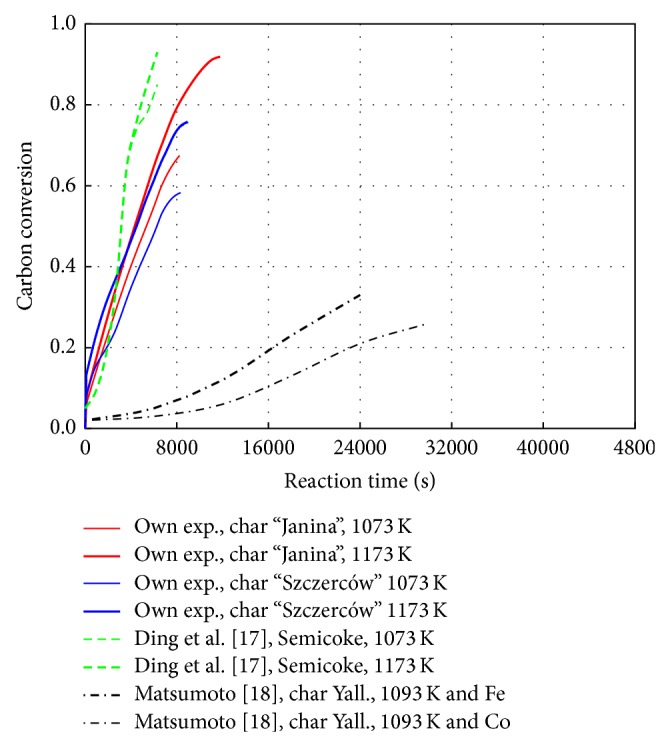
Influence of temperature on carbon conversion rate.

Effects of the hydrogasification isothermal stage temperature (1073, 1173 K) on the char conversion rate at 6 MPa for the “Janina” char are presented in Figure 2. The findings were compared to the results obtained by Ding et al. [17] for the “Inner Mongolia Semicoke” at the same temperatures and at the pressure of 5 MPa, as well as to the experiments performed by Matsumoto [18] for the “Yallourm” char at 1093 K and 0.1 MPa with a catalyst: iron (0.80% Fe) and cobalt (0.92% Co). For the “Janina” char, the maximum conversion rate at 1073 K was comparable to that obtained by Ding et al., while at 1173 K, it was lower by 18%. With the additive of iron, the results of catalytic hydrogasification obtained by Matsumoto were approximately 2-fold lower than the findings for the “Janina” char, while with the cobalt additive, they were approximately 2.5-fold lower. The conversion rates were clearly affected by low pressure despite the use of catalysts. Similar results to Matsumoto's findings were obtained by Lee et al. [19] for the “Datong” coal, which was gasified with hydrogen at 973 K and 6 MPa.

**Figure 3:**
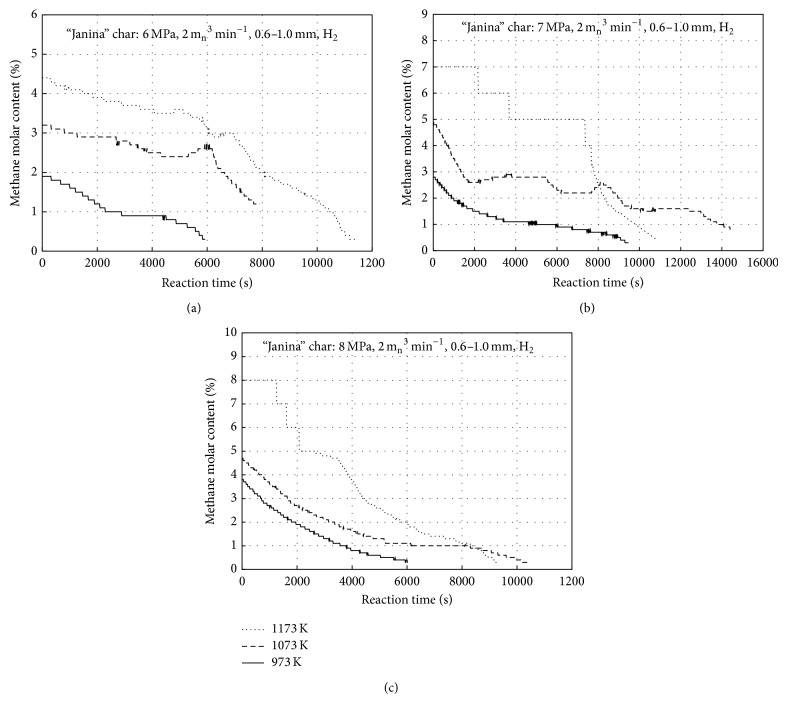
Measured molar content of the outflow gas methane versus time for “Janina” char at 1173, 1073, and 973 K for (a) 6 MPa, (b) 7 MPa, and (c) 8 MPa.

**Figure 4:**
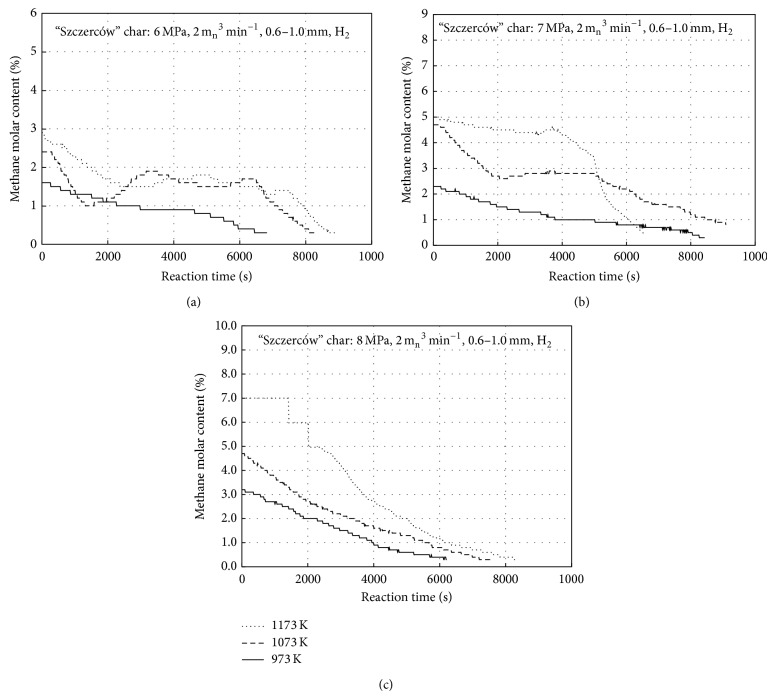
Measured molar content of the outflow gas methane versus time for “Szczerców” char at 1173, 1073, and 973 K for (a) 6 MPa, (b) 7 MPa, and (c) 8 MPa.

Effects of the hydrogasification isothermal stage temperature (973, 1073, and 1173 K) and pressure (6, 7, and 8 MPa) are presented in Figures 3(a) to 3(c) for the “Janina” char and in Figures 4(a) to 4(c) for the “Szczerców” chars, respectively. For each plot, a hydrogasification onset was assumed to be the moment when the maximum methane concentration was achieved for the process temperature. A stepped shape of the lines in the figures is a result of the applied gas analyser resolutions: for 0–5% methane, the measurement accuracy was 0.1% while for concentrations above 5%, the accuracy was 1%. The highest CH_4_ molar fractions (approximately 8% and 7% for the “Janina” and “Szczerców” chars, resp.) were reported for 1173 K and 8 MPa, while the lowest values (approximately 2% and 1.6% for the “Janina” and “Szczerców” chars, resp.) were recorded at 973 K and 6 MPa. The temperature rise by 200 K and the pressure rise by 2 MPa allowed for obtaining more than twofold higher CH_4_ fractions.

### 3.2. Char Reactivity

For industrial applications, important technological parameters are char reactivity in the hydrogasification process and its relationship with the solid carbon conversion rate. In the literature, experimental facts suggestive of strong char reactivity reduction during hydrogasification at high conversion rates [1, 2, 20] can be found. Such reduction, which impedes achievement of a solid carbon high conversion rate, has resulted in a search of industrial technological solutions that will ensure maintaining major hydrogasification benefits. This is a basis for a process involving two reactors [21]: a reactor for coal hydropyrolysis and a reactor for char gasification.

The process is aimed at synthetic natural gas (SNG) and benzene/toluene/xylene mixture (BTX) production with the energy performance of approximately 77%, which is far higher than the 63% value obtained for the SNG production technology based on methanation of synthesis gas obtained by coal gasification with steam and oxygen [22]. The process parameters, basically pressure and temperature, are selected based on the structures of expected final products. The hydropyrolysis reaction at a moderate pressure of approximately 3 MPa and a temperature of up to 1143 K suggests a potential for achieving up to 88% energy performance with two final products: fuel gas (H_2_, CO, and CH_4_) and light oils [21]. For only SNG or BTX production, a higher process pressure of up to 8 MPa is necessary, which results in process energy performance reduction to approximately 77%.

**Figure 5:**
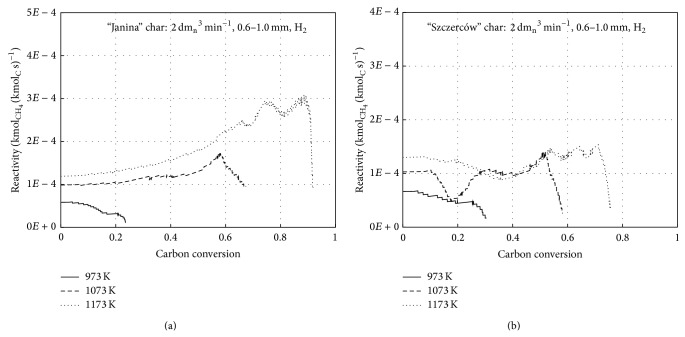
Hydrogasification reactivity versus carbon conversion time for (a) “Janina” and (b) “Szczerców” char.

Reactivity of chars, expressed as a ratio of produced methane molar stream to a current number of moles of carbon remaining in the solid state in the reactor, was determined based on the experiments performed for the “Janina” and “Szczerców” coal chars. In Figure 5, results for 6 MPa at three temperatures, 973 K, 1073 K, and 1173 K, are presented. The figures show that initially, hydrogasification proceeded with almost stable reactivity (or even mildly increasing), while in the finale stage, a rapid reactivity drop was observed. For the highest temperature of 1173 K, the rapid reactivity drop began at approximately 70% and from approximately 90% carbon conversion rate for the “Szczerców” and “Janina” chars, respectively. The investigations do not confirm previous observations of gradual char reactivity reduction. Mühlen [2] observed a continuous, relatively mild reactivity drop during hydrogasification at 4 MPa. At the coal conversion degree of approximately 90%, the reactivity drop was about 75% of the initial value. On the other hand, Anthony et al. [9] observed a rapid reactivity drop as early as at the 10% C conversion degree during char hydrogasification at 873 K, 998 K, and 3.3 MPa. The results presented in Figure 5 show that for the lowest investigated temperature, 973 K, a reactivity drop can be assumed to start as early as from the hydrogasification onset. However, for 1073 K and 1173 K in particular, a rapid reactivity drop did not occur until the carbon conversion rate was above 60% and 80%, respectively. This means that, to obtain a high degree of carbon conversion, a temperature of approximately 1200 K is necessary during char hydrogasification.

### 3.3. Coal Hydrogasification

The “Janina” and “Szczerców” coal hydrogasification schedule involved experiments with 10 g samples at 973, 1073, and 1173 K; 8 MPa; and hydrogen flow rate of 0.5 dm^3^/min. During the coal hydrogasification experiments, no transfer of liquid tarry substances with the process gas or adhesion to the gas outflow channels were observed, which means that the tarry products of the devolatilisation stage in the hydrogen atmosphere were mainly conversed to the C_1_–C_3_ gaseous form. Deep conversion of the tarry substances resulted from relatively long retention time of the gaseous phase (approximately 6 s) in the high temperature zone. Dobner et al. [23] demonstrated that, under similar coal hydrogasification conditions, a 3 s stay of the gaseous phase in the reactor was sufficient for complete conversion of the tarry substances to gaseous hydrocarbons.

Results of maximum molar fraction measurements for methane contained in the process gas versus time for the “Janina” and “Szczerców” coals at the hydrogasification isothermal stage temperatures (1173 K and 973 K) against hydrogasification of their chars are presented in Table 2. Maximum methane fractions in the process gas were observed in the initial hydrogasification stage and they were temperature-dependent. For the maximum temperature of 1173 K, in the coal hydrogasification case, the methane fraction was approximately 40% lower than for the char. For 973 K, coal hydrogasification resulted in a gas of higher methane content than in the case of char hydrogasification. This relationship was a result of hydropyrolysis and hydrogasification overlap. The hydrogasification process at 973 K was more than twofold slower, so its contribution to methane production at lower temperatures was less important compared to hydropyrolysis, while at high temperatures, the situation was reversed, due to intense methane production during char hydrogasification. A significant reaction may be development of the inner surface of char particles resulting from their preparation at 1273 K, which was higher than the hydrogasification temperatures of 973 K or 1173 K.

### 3.4. Char Hydrogasification in the H_2_ + CO_2_ Atmosphere

A technological process of hydrogasification requires elimination of carbon dioxide following synthesis gas-contained CO conversion. The final hydrogen purity affects the process economy and has clear technological consequences for the hydrogasification stage. To check this, hydrogasification in a carbon dioxide-diluted hydrogen atmosphere was investigated for the H_2_ + 10% CO_2_ mixture. The experiments were performed at the highest pressure of 8 MPa and for one gas flow rate of 0.5 dm_n_^3^/min at three temperatures: 973, 1073, and 1173 K. As the maximum methane concentrations obtained during the experiments were higher than those obtained during gasification in hydrogen, a role of methane generation during a homogenous reaction in the H_2_ + 10% CO_2_ mixture was investigated. For this purpose, a carrier gas was delivered to the reactor without a char sample under the same thermal conditions.

Results of measurements of methane molar fraction in the process gas for the “Janina” char are presented in Table 3. The highest (approximately 20%) and the lowest (approximately 8-9%) CH_4_ molar fractions were reported for 1173 K and 973 K, respectively. Under the comparable conditions of char hydrogasification in pure hydrogen, the obtained maximum methane concentrations were markedly lower. A fraction of methane generated in homogenous reactions of CO_2_ and H_2_ was about 40–50% of the total content at the highest recorded concentrations for all three investigated cases (as apparent from the analysis of Table 3).

**Figure 6:**
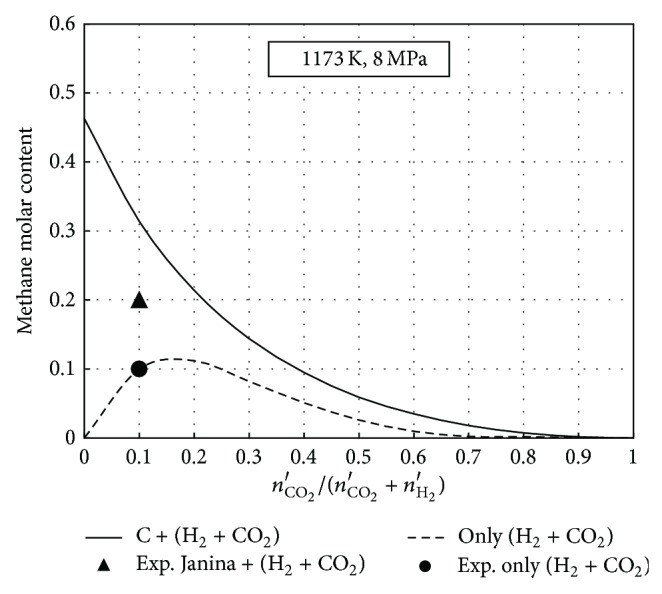
CH_4_ molar fraction in the hydrogasification products with respect to CO_2_ content in the inlet gas.

In the experiments of hydrogasification in the H_2_ + 10% CO_2_ atmosphere, generation of significant CH_4_ amounts in the reactor without solid carbon was demonstrated. As shown in Table 3, the methane fraction at the reactor outflow point may reach up to approximately 20% at 8 MPa and 1173 K. To assess technological consequences of CO_2_ effects on the hydrogasification process, an equilibrium analysis of the products leaving the reactor was performed for 1173 K and 8 MPa process parameters. It was assumed that the solid reagent was char (C), while the calculations were performed for the equal to one molar ratio of the inlet gas amounts to the C. The molar fraction of CO_2_ in the inlet gas was analysed within the 0 to 1 range and compositions of hydrogasification products at the chemical equilibrium were determined for a six-component system: H_2_, CO_2_, CO, H_2_O, CH_4_, and C. In Figure 6, the equilibrium fraction of methane in the char hydrogasification gas with respect to the initial CO_2_ content in the carrier gas (H_2_ + CO_2_) is presented. The plot analysis shows that the CO_2_ fraction in the carrier gas markedly affects the equilibrium methane content in the produced process gas: for only 20% CO_2_ in the carrier gas, the CH_4_ fraction was reduced by a half. Moreover, a line representing methane content in the gas leaving the reactor without a char sample is presented. In this case, methane is produced during homogenous reactions in a five-component system: H_2_, CO_2_, CO, H_2_O, and CH_4_. While comparing the experimental results for 1173 K in Table 3 to the lower line in Figure 6, it is clearly seen that the gas leaving the reactor without a char sample reached the equilibrium-close state, while the char hydrogasification reaction was approximately 10% higher for the CH_4_ molar fraction. It means that 10% CO_2_ in the hydrogen-rich inlet gas enhanced the hydrogasification process, which should have beneficial technological consequences.

## 4. Conclusions

Consider the following:

1. Essential parameters for coal hydrogasification in pure hydrogen with respect to the process performance are pressure, temperature, and a flow rate of hydrogen delivered to the reaction.
2. A high degree of char hydrogasification can be achieved by performing the process at approximately 1200 K.
3. The hydrogasification process at 973 K is more than twofold slower than at 1173 K, so hydrogasification contribution to methane production at lower temperatures is less important compared to hydropyrolysis while at high temperatures, the situation is reversed due to intense methane production during char hydrogasification.
4. Reactivity of the “Szczerców” lignite char is comparable to reactivity of the “Janina” coal within the investigated technical parameter range.
5. Under similar conditions of char hydrogasification in the hydrogen atmosphere with the 10% carbon dioxide additive, markedly higher maximum methane concentrations were obtained than those obtained during hydrogasification in pure hydrogen.
6. The additive of approximately 10% carbon dioxide to hydrogen markedly enhances the process of char hydrogasification.

## Figures and Tables

**Table 1:** Characteristics of coals used.

Coal	W^a^, %	V^a^, %	A^a^, %	C^a^, %	H^a^, %	S^a^, %	N^a^, %
“Janina” coal	10.21	32.41	9.46	62.73	3.94	1.45	0.84
“Szczerców” lignite	12.97	37.53	23.07	42.63	3.21	2.48	0.34

^a^Analytical state.

**Table 2:** Maximum methane molar content in the outflow gas in hydrogasification at 8 MPa and hydrogen flow rate of 0.5 dm_n_^3^/min.

Sample	Methane molar content, %		
	973 K	1073 K	1173 K
“Janina”			
Coal	4.3	4.9	5.9
Char	3.8	4.7	10.0
“Szczerców”			
Coal	2.9	3.2	3.7
Char	1.6	3.4	6.1

**Table 3:** Comparison of the maximum molar methane content in the outflow gas.

Temperature	H_2_	H_2_ + 10% CO_2_
973 K	3.8%	9%
1073 K	4.7%	13%
1173 K	10%	20%
